# Enriched oxygen improves age-related cognitive impairment through enhancing autophagy

**DOI:** 10.3389/fnagi.2024.1340117

**Published:** 2024-02-14

**Authors:** Shengyuan Wang, Bengang Chen, Minghao Yuan, Shu Liu, Haixia Fan, Xu Yang, Qian Zou, Yinshuang Pu, Zhiyou Cai

**Affiliations:** ^1^Chongqing Medical University, Chongqing, China; ^2^Chongqing Institute Green and Intelligent Technology, Chinese Academy of Sciences, Chongqing, China; ^3^Chongqing School, University of Chinese Academy of Sciences, Chongqing, China; ^4^Department of Neurology, Chongqing General Hospital, Chongqing, China

**Keywords:** hyperbaric oxygen therapy, autophagy, AMPK-mTOR pathway, tau hyperphosphorylation, cognitive impairment

## Abstract

Age-related cognitive impairment represents a significant health concern, with the understanding of its underlying mechanisms and potential interventions being of paramount importance. This study aimed to investigate the effects of hyperbaric oxygen therapy (HBOT) on cognitive function and neuronal integrity in aged (22-month-old) C57BL/6 mice. Male mice were exposed to HBOT for 2 weeks, and spatial learning and memory abilities were assessed using the Morris water maze. We employed transcriptome sequencing and Gene Ontology (GO) term enrichment analysis to examine the effects of HBOT on gene expression profiles, with particular attention given to synapse-related genes. Our data indicated a significant upregulation of postsynapse organization, synapse organization, and axonogenesis GO terms, likely contributing to improved cognitive performance. Moreover, the hyperphosphorylation of tau, a hallmark of many neurodegenerative diseases, was significantly reduced in the HBO-treated group, both *in vivo* and *in vitro*. Transmission electron microscopy revealed significant ultrastructural alterations in the hippocampus of the HBOT group, including an increase in the number of synapses and the size of the active zone, a reduction in demyelinated lesions, and a decreased number of “PANTHOS.” Furthermore, Western blot analyses confirmed the upregulation of PSD95, BDNF, and Syn proteins, suggesting enhanced synaptic plasticity and neurotrophic support. Moreover, HBOT increased autophagy, as evidenced by the elevated levels of Beclin-1 and LC3 proteins and the reduced level of p62 protein. Finally, we demonstrated that HBOT activated the AMPK-mTOR signaling pathway, a critical regulator of autophagy. Notably, our findings provide novel insights into the mechanisms by which HBOT ameliorates age-related cognitive impairment, suggesting the potential therapeutic value of this approach.

## Introduction

Aging is characterized by a progressive loss of physiological integrity, which refers to the gradual decline and deterioration of various biological functions and systems in the body. This process occurs over time and is associated with manifold alterations at the cellular, tissue, and organismal levels (Campisi et al., 2019; López-Otín et al., 2023). Notably, the aging process significantly impacts the nervous system, making it one of the most affected systems (Bishop et al., 2010). Consequently, with the global aging trend, age-related cognitive impairment has emerged as a significant health concern (Livingston et al., 2017). Thus, identifying effective interventions for enhancing cognitive function in aging individuals is paramount.

Hyperbaric oxygen therapy (HBOT) involves administering 100% oxygen at pressures exceeding one atmosphere absolute (ATA), and it is now widely recognized as an effective treatment for various conditions (Tibbles and Edelsberg, 1996; Al-Waili et al., 2005; Shapira et al., 2018). This non-invasive, safe, and frequently used therapy is gaining traction for new potential uses, including combating aging and related diseases (Balasubramanian et al., 2021). A recent study has also revealed that HBOT mitigated oxidative stress, inflammation, apoptosis, amyloid-beta deposition, and cholinergic system dysregulation, providing neuroprotection (Chen et al., 2017; Shwe et al., 2021). Furthermore, HBOT has been found to have a relationship with autophagy (Fang et al., 2019), a cellular process involved in the degradation and recycling of damaged or unnecessary cellular components.

Autophagy is a universally preserved cellular mechanism crucial for maintaining cellular balance. It achieves this by breaking down and recycling damaged proteins and organelles (Dikic and Elazar, 2018). It is also essential for ensuring cellular quality control and adaptation to various stress conditions (Mizushima and Komatsu, 2011). Autophagy plays a pivotal role in various age-related neurodegenerative diseases, including Alzheimer’s disease, Parkinson’s disease, and Huntington’s disease, by regulating intracellular waste clearance, cellular survival, and metabolism (Menzies et al., 2017). Adenosine monophosphate-activated protein kinase (AMPK) and mammalian target of rapamycin (mTOR) are pivotal signaling pathways that play crucial roles in regulating cellular metabolism, energy homeostasis, and autophagy (Herzig and Shaw, 2018; Xie et al., 2023). Importantly, HBOT has been found to have a relationship with autophagy. One study indicates that HBOT promotes mitophagy and relieves pain by mediating the AMPK signaling pathway in rats with neuropathic pain (Kun et al., 2019). Another study demonstrated that HBOT activates SIRT1, enhancing autophagy and improving motor dysfunction in rats with traumatic spinal cord injury (Chen et al., 2023). However, the specific mechanism between HBOT and autophagy remains unclear.

Based on the understanding of the roles played by AMPK, mTOR, and autophagy in maintaining cellular and organismal homeostasis (Alers et al., 2012), it has been hypothesized that HBOT may enhance cognitive function in aging individuals by activating the AMPK/mTOR-mediated autophagic pathway. In this study, we employed an established murine senescence model and exposed the rodents to sessions of HBOT. We evaluated cognitive function via behavioral assessments and analyzed the activation of AMPK, mTOR, and autophagy markers in the brain tissue of mice. Additionally, HBOT may alleviate hyperphosphorylation of the tau protein and synaptic dysfunction. It is essential to comprehend the potential advantages of HBOT in enhancing cognitive function among aging individuals, as this knowledge can aid in developing effective strategies to combat age-related cognitive decline. Furthermore, exploring the involvement of the AMPK/mTOR-mediated autophagy pathway in this process may yield valuable insights into the underlying mechanisms and pave the way for targeted therapeutic interventions.

## Materials and methods

### Animal models

**Figure 1:**
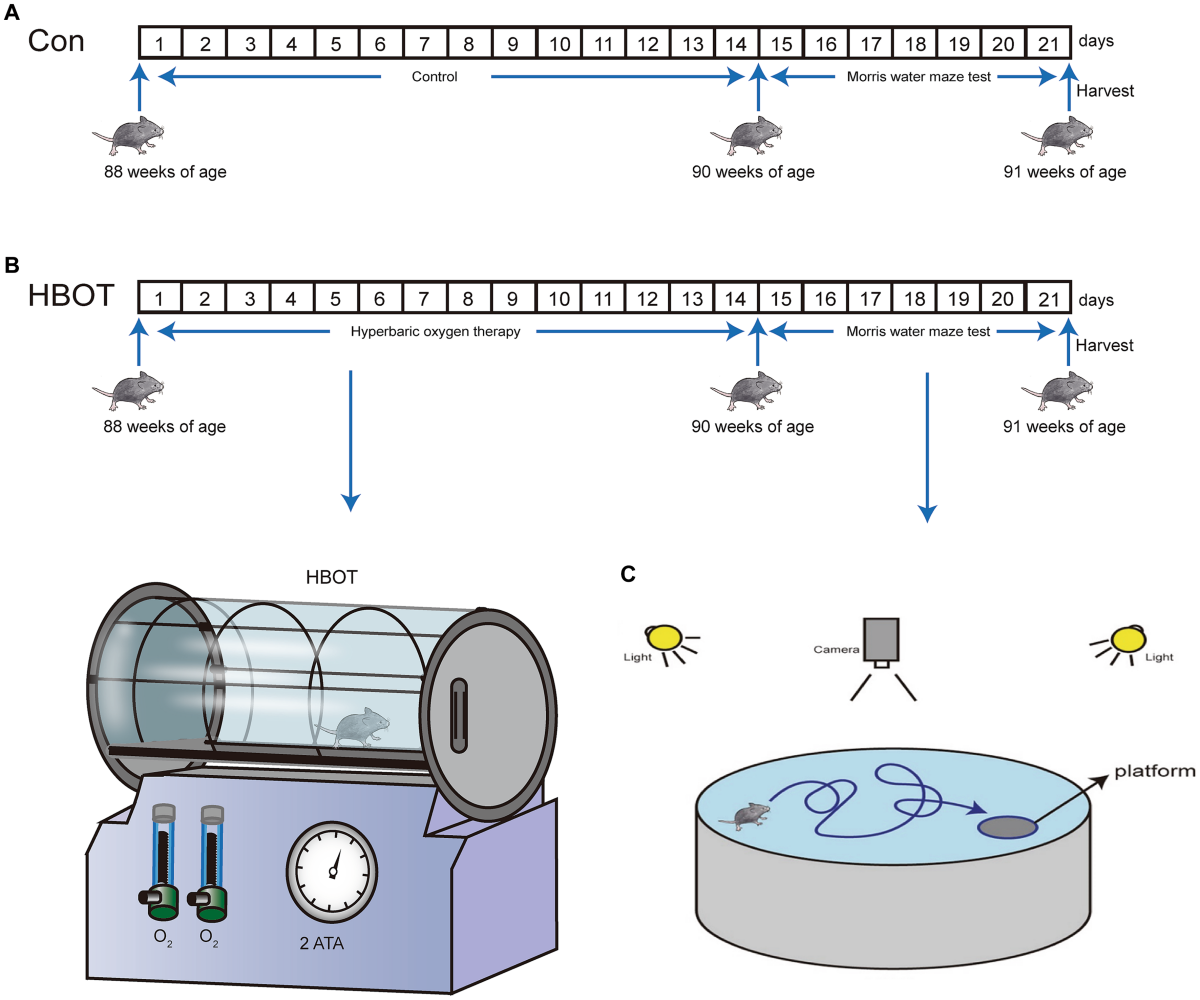
Enhanced representation of the experimental model for HBOT and its impact on behavior in mice. **(A)** Time progression diagram of mice experiment. **(B)** Schematic representation of HBOT in mice models. **(C)** Schematic illustration of the experimental setup for the Morris water maze test in mice.

The 22-month-old male C57BL/6 mice utilized in the study were acquired from Chengdu Dossy Experimental Animals Co., Ltd., located in Chengdu, China. And each animal was kept in a 12 h light/12 h dark cycle. The Research Ethics Committee of Chongqing Medical University approved all animal experiments involving animals. Experimental methods followed the National Institutes of Health Guide for Care and Use of Laboratory Animals. Mice were free to access drinking water and food. When the mice were 22 months old, they were subjected to HBOT for 2 weeks and then to the Morris water maze (MWM) test for 1 week (Figure 1). After completing the behavioral tests, the mice were euthanized via decapitation to minimize any potential indirect effects on tau phosphorylation due to hypothermia or anesthesia (Bretteville et al., 2012). The brains were swiftly extracted and rinsed with chilled normal saline. Subsequently, the samples were stored at −80°C or kept at 4°C for additional analysis involving techniques like liquid chromatography–tandem mass spectrometry (LC–MS/MS), transmission electron microscopy (TEM), and Western blotting. This ensured the preservation of brain tissue integrity and allowed for accurate assessment of tau phosphorylation and other molecular markers.

### Hyperbaric oxygen therapy

HBOT was administered to the mice using a hyperbaric chamber. The mice were exposed to a pressure of 2.5 ATA for 90 min (Tibbles and Edelsberg, 1996). Prior to the therapy, 15 min of pressurization and depressurization were provided to allow the mice to acclimate to the pressure changes. The HBOT procedure involved a gradual increase in pressure over 10–15 min to reach 2.5 ATA in a 100% oxygen environment. The mice were then exposed to the sustained pressure of 2.5 ATA for 90 min, followed by a 15-25-min decompression phase. The HBOT protocol used in this study was adapted from a previous *in vitro* study (Yi et al., 2018).

### Cell cultivation methods

The Neuro-2a (N2a) mouse neuroblastoma cells were acquired from Procell Life Science & Technology Co., Ltd., based in Wuhan, China. These cells were cultured in Dulbecco’s Modified Eagle’s Medium (DMEM-F12) procured from Gibco, based in Carlsbad, CA, United States, and supplemented with 10% fetal bovine serum (FBS) from Invitrogen, located in Irvine, CA, United States. This medium was further supplemented with antibiotics: 100 U/mL penicillin and 100 μg/mL streptomycin. The N2a cell culture was maintained under a humidified condition, incorporating 5% CO_2_ at a steady temperature of 37°C.

### Morris water maze

When the 22-month-old mice in the experimental group received 2 weeks of HBOT, the control group and the experimental group mice were verified by behavioral experiments. The spatial learning and memory abilities of the two groups were analyzed using the Morris Water Maze test. There was a circular pool with a diameter of 120 cm and a height of 40 cm, filled with 25°C ± 1°C water. With the addition of non-fat milk, the water became opaque and was divided into four equal quadrants (I, II, III, IV). There was a hidden square platform (8 cm in diameter) 1 cm below the water surface in the middle of quadrant I. A digital camera was placed 1.7 m above the center of the maze to record trials. During the first consecutive 5 days of the experiment, the mice were exposed to water for 4 sessions of the swim. In each experiment, the mice entered the water from each of the four quadrants and measured the time it took to reach the platform (escape latency). If the mice failed to reach the platform within 60 s. The experimenter will place the mice directly on the platform and hold them for 30 s. The average time of the 4 sessions of the swim represented the escape latency time of individual mice. Calculate the daily average for each group day-by-day. On the sixth day of the experiment, the platform was removed for probe test. In the experiment, the mice entered the water from the third quadrant and were allowed to search for a platform in the water for 60 s. The swimming trajectory data were digital video-recorded and analyzed by the ANY-maze tracking software (Stoelting Co., Wood Dale, United States). The data analyzed encompassed several parameters: total time and length of swimming, time and length of swimming in designated quadrants, and the number of times mice crossed the target platform area, all within a period of 60 s.

### Western blotting

Total proteins of brain tissue of mice were extracted by RIPA lysis Buffer containing 1% phenylmethylsulfonyl fluoride (PMSF) for 30 min on ice. The protein concentration was measured by a BCA protein assay kit (Beyotime, shanghai, China). Fifty micrograms (50 μg) of protein from each sample were separated on 8–12.5% SDS-PAGE and transferred to polyvinylidene difluoride (PVDF) membranes. Following a 2-h blocking period at room temperature using a solution of 5% non-fat dry milk in TBST buffer, the membranes were exposed to a series of primary antibodies at a temperature of 4°C overnight. These antibodies included those against proteins like brain-derived neurotrophic factor (BDNF) (Proteintech Group, Inc., cat. 28,205-1-AP, used at a 1:1,000 dilution), postsynaptic density protein 95 (PSD95) (Proteintech Group, Inc., cat. 20,665-1-AP, used at a 1:1,000 dilution), and synaptophysin (Syn) (Proteintech Group, Inc., cat. 17,785-1-AP, used at a 1:20,000 dilution). Other antibodies targeted Tau46 (Cell Signaling Technology, cat. #4019), phospho-tau at positions Thr205, Thr181, and Ser404 (Cell Signaling Technology, cat. #49561, #12885, #20194, respectively), AMPKα (Cell Signaling Technology, cat. #2532), phospho-AMPKα at position Thr172 (Cell Signaling Technology, cat. #2535), mTOR (Cell Signaling Technology, cat. #2983), phospho-mTOR at position Ser2448 (Cell Signaling Technology, cat. #5536), SQSTM1/p62 (Cell Signaling Technology, cat. #23214), LC3A/B (Cell Signaling Technology, cat. #58139), Beclin-1 (Cell Signaling Technology, cat. #3495), GAPDH (Beyotime, cat. AF0006), and β-actin (Beyotime, cat. AF5003). Following washes in TBST buffer, the membranes were then subjected to incubation with secondary antibodies that included goat anti-rabbit (Beyotime, China, cat. A0208) and goat anti-mouse (Beyotime, China, cat. A0192), both at a dilution of 1:1,000. Thereafter, the blots were incubated with corresponding secondary antibody for 1 h at room temperature. Following triple rinses with TBST, the membranes were treated with ECL solution (Beyotime, Shanghai, China) for detection and imaged using a Tanon system (Shanghai, China). Band intensity was quantified using ImageJ software.

### RNA sequencing

RNA Extraction: three fresh brain tissues (6 mice in two groups) were collected from animals in each group with a saline perfusion for RNA sequencing. All tissues harvested were frozen in liquid nitrogen and stored at 80°C until use. To extract the RNA from the mouse brain tissue, an Invitrogen Trizol reagent kit was used (Carlsbad, CA, United States). Furthermore, Nanodrop One (Thermo Fisher Scientific, MA, United States) and Qubit 2.0 were used simultaneously to measure RNA quantities. The integrity of RNA was determined using an Agilent 2,100 Bioanalyzer (Agilent Technologies, Palo Alto, CA, United States).

RNA sequencing: mRNA was purified from total RNA using Oligo (dT)-attached magnetic beads. After that, we fragmented the mRNA by adding the fragmentation buffer. The first strand of cDNA was synthesized using six-base random hexamers with mRNA as a template. Next, dNTPs, buffer, and DNA polymerase I were added to produce the second strand of cDNA. Purification of double-stranded cDNA was then achieved using AMPure XP beads. Following purification, cDNA was end-repaired and “a-tailed” for adapter ligation, and AMPure XP beads were used to select fragment size. PCR was used to enrich cDNA fragments of suitable size to obtain the final cDNA library.

Sequencing data preprocessing and analysis: The quality of sequencing data in raw FASTQ files was evaluated with FastQC.^1^ High-quality clean reads were mapped to ribosome RNA (rRNA) to identify residual rRNA reads. Reads obtained by the sequencers were filtered to remove adapters, reads containing more than 10% unknown nucleotides, and reads containing more than 50% low-quality bases. HISAT2 was used to map the remaining trimmed sequences (clean reads) to the designated genome using the default settings. Using the mapping results, read counts for each gene were calculated. The two groups’ gene expressions were compared with DESeq R software,^2^ considering the length and number of genes. Differentially expressed genes (DEGs) were based on normalized data. After that, the package limma applied an empirical Bayesian shrinkage method to obtain a *t*-test statistic and its *p*-value. Benjamini-Hochberg correction was applied to account for multiple tests. As significant DEGs, genes with an absolute fold change ≥ 2-fold and FDR less than 0.05 were considered. Those genes were used for the enrichment analysis of the Kyoto Encyclopedia of Genes and Genomes (KEGG).^3^ Furthermore, in gene set enrichment analyses (GSEA), a threshold for differential expression of genes is not required to be specified, and there is a larger functional range.

### Immunofluorescence

Mice, anesthetized and grouped (*n* = 3 per group), underwent perfusion with ice-cold 0.1 M phosphate-buffered saline (PBS). The mice’s brains were harvested after they were perfused with 4% ice-cold paraformaldehyde. The entire brain was submitted to 24-h fixation in 4% paraformaldehyde at 4°C. The fixed brain tissues were dehydrated in gradient ethanol and transparent with xylene before being embedded in paraffin. Then sections of paraffin were cut using a microtome (HM 340E, 9 Thermo Scientific) at a thickness of 3 mm in a sagittal plane. Following deparaffinization and rehydration, by boiling in a citric acid buffer in the microwave (high fire for 5 min, medium fire for 15 min), we were able to retrieve antigens from paraffin sections.

For the process of DAPI staining and immunofluorescence, tissue sections were first washed with PBS and then treated with 5% normal goat serum (NGS) for an hour at ambient temperature. Subsequently, they were incubated overnight at 4°C with primary antibodies against SQSTM1/p62 (dilution 1:1,000, catalog #23214, Cell Signaling Technology) and LC3A/B (dilution 1:1,000, catalog #58139, Cell Signaling Technology). After washing thrice with PBS, the sections were exposed to the secondary antibody (dilution 1:300, Alexa Fluor 488-conjugated goat anti-rabbit IgG, catalog ZF-0516, ZSGB-BIO, China) for 1 h at 37°C. Nuclei staining was performed using DAPI (Beyotime). Post washing with PBS, the coverslips were mounted on slides using Antifade Mounting Medium (Beyotime), and fluorescence imaging was conducted using a NEXCOPE microscope (model NE900, United States).

### Transmission electron microscopy

Mice were deeply anesthesia by injecting 100 μg pentobarbital intraperitoneally after they had completed the MWM. Anesthetized mice were perfused through the left ventricle with saline and after washing away the blood, followed by a mixture of 2.5% glutaraldehyde and 4% paraformaldehyde for fixing brain tissue *in situ*. On ice, hippocampus tissues were isolated and cut into 1 mm^3^ cubes. Tissue specimens were fixed with 4% glutaraldehyde for 4 h, followed in pre-cooled PBS buffer (PH = 7.2) for 15 min three times. Next, the specimens were post-fixed in 1% osmium tetroxide (GP18456, Leica) on ice for 2 h and washed three times with pre-cooled PBS buffer (PH = 7.2) for 15 min each. The dehydration tissue samples were dehydrated with 30, 50, 70, 80, and 90% acetone, respectively, for 30 min, followed by 100% acetone for 30 min for three times. The embedding agent was then infiltrated into the samples for 3 h at room temperature. Then, the tissue samples were placed in a mixture of acetone/embedding agent (1:1) at 37°C for 1 h, acetone/embedding agent (1:2) at 37°C overnight for infiltration and then placed in pure embedding agent at 37°C for 5–8 h. The embedded samples were incubated at 37°C in a dry oven for 24 h, and then in 60°C dry oven for 48 h to form a solid embedding block. Ultrathin sections (50 nm) were prepared using an ultramicrotome (EM UC7, Leica), stained with uranyl acetate and lead citrate and examined with electron microscopy (JTM-1400FLASH, Japan).

### SA-β-gal cytochemical staining

Cytochemical staining for the activity of senescence-associated β-galactosidase (SA-β-gal) was conducted following established procedures using a commercial SA-β-galactosidase assay kit (Beyotime, C0602) following the manufacturer’s guidelines.

In this method, mouse groups (3 mice per group) were humanely euthanized using tricaine. The specimens were then embedded in Tissue-Tek O.C.T. compound and preserved at −80°C. Cryostat-generated sections, 10 micrometers thick, were placed on slides. Subsequently, the slides were air-dried, briefly immersed in PBS at ambient temperature for 5 min, and fixed in a solution for 30 min. Following fixation, the slides underwent a PBS wash, and each was treated with 1 mL of β-galactosidase staining solution.

This staining occurred overnight at 37°C, displaying blue senescence staining in the cytoplasm of aged cells, observed under 200× magnification. Quantitative assessment of SA-β-gal-positive cells involved selecting and counting four random fields per section using ImageJ software.

### Statistical analyses

The randomization of mice was conducted using the random number generator function (RANDBETWEEN) in Microsoft Excel. For data analysis, we utilized the GraphPad Prism Software, version 9. To examine the normal distribution of the data, we implemented the Shapiro–Wilk test. Subsequently, the data difference between the two groups was evaluated using Student’s *t*-test, Mann–Whitney U test, or repeated measures analysis of variance. Data are presented as mean ± standard deviation (SD), with significance levels designated as *p < 0.05, **p < 0.01, and ***p < 0.001.

## Results

### HBOT improved cognitive dysfunction in aged C57BL/6 mice

**Figure 2:**
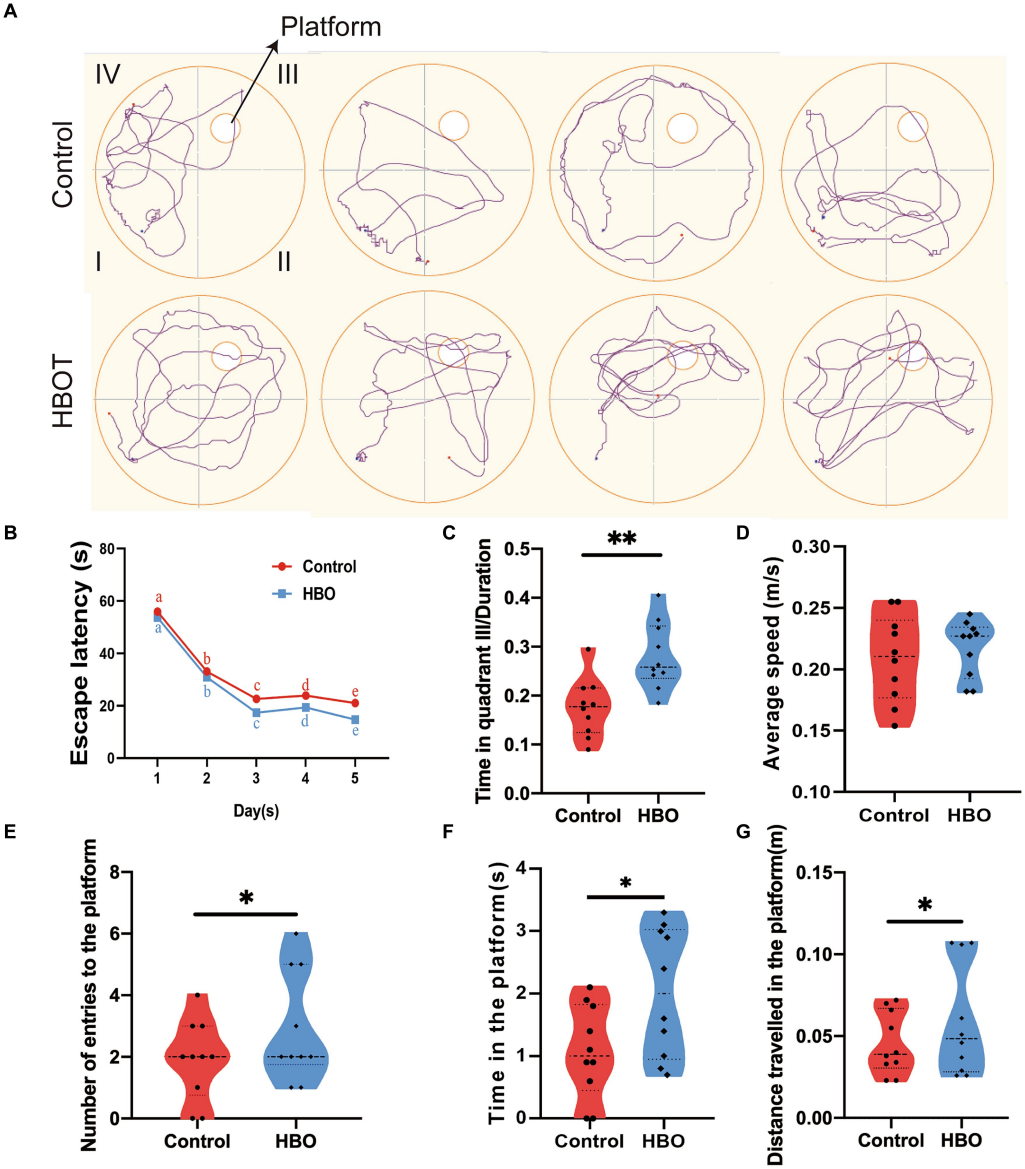
Hyperbaric oxygen improves spatial learning and memory in aged C57BL/6 mice. Experiment and timeline procedure. Ninety-week-old C57BL/6 mice were divided into Control and HBO groups. Swimming traces of mice in the probe trial are presented in **(A)**. HBO led to significant changes in average escape latency. The letter notation **(a, b, c, d, e)** represents the comparisons of differences between time points, and no significant differences were observed between groups **(B)**, number of entries to the platform **(E)**, time in the platform **(F)**, distance travelled in the platform **(G)**, and frequency in the targeted quadrant **(C)** during the whole trial compared with those of Control. There was no significant difference in the swimming speed between Control and HBO groups **(D)**. *n* = 10/group, two-sample *t*-test. Mean ± SEM, *p < 0.05, **p < 0.01, compared with Control group. HBO, hyperbaric oxygen.

MWM results demonstrated a progressive reduction in escape latency for both groups over time, indicative of learning. However, after 2 weeks of HBOT, the control group exhibited longer escape latencies compared to the HBOT group on both the 4th and 5th days (*p* < 0.05 for both days) (Figure 2B). Figure 2A displays the swimming paths of the control and HBO mice during the spatial exploration task, following the hidden platform trials. The control group displayed significant differences from the HBO group regarding time spent on the platform, the distance covered to reach the platform, and the frequency of entries into the platform quadrant during probe trials (Figures 2E–G). Furthermore, control mice allocated less time to the targeted quadrant (quadrant III) compared to the HBO mice (Figure 2C). No significant differences were observed in swimming speed between the groups (Figure 2D), thereby negating the possibility of swimming ability influencing the MWM performance differences. Notably, our findings suggest that a 14-day HBOT regime enhances spatial learning and memory in naturally aged mice, as evidenced by the water maze experiment. Moreover, the treated group exhibited shorter escape latency, increased time spent on the platform, more entries onto the platform, greater distance traveled toward the platform, and increased time spent in the targeted quadrant compared to the control group. These results suggest HBOT enhances spatial memory and learning ability in aged mice.

### Transcriptomic analysis reveals enhanced synaptic function and plasticity in aged C57BL/6 mice brain following HBOT

To investigate the specific effects of HBOT on the aging mouse brain, we performed eukaryotic transcriptome sequencing analysis on brain tissues from two groups of aged C57BL/6 mice (*n* = 3). The transcriptome sequencing analysis revealed significant alterations in gene expression profiles between the HBO treatment group and the control group of aged mice. From this analysis, we gained insights into the specific effects of HBO treatment on the aging brain at the molecular level.

**Figure 3:**
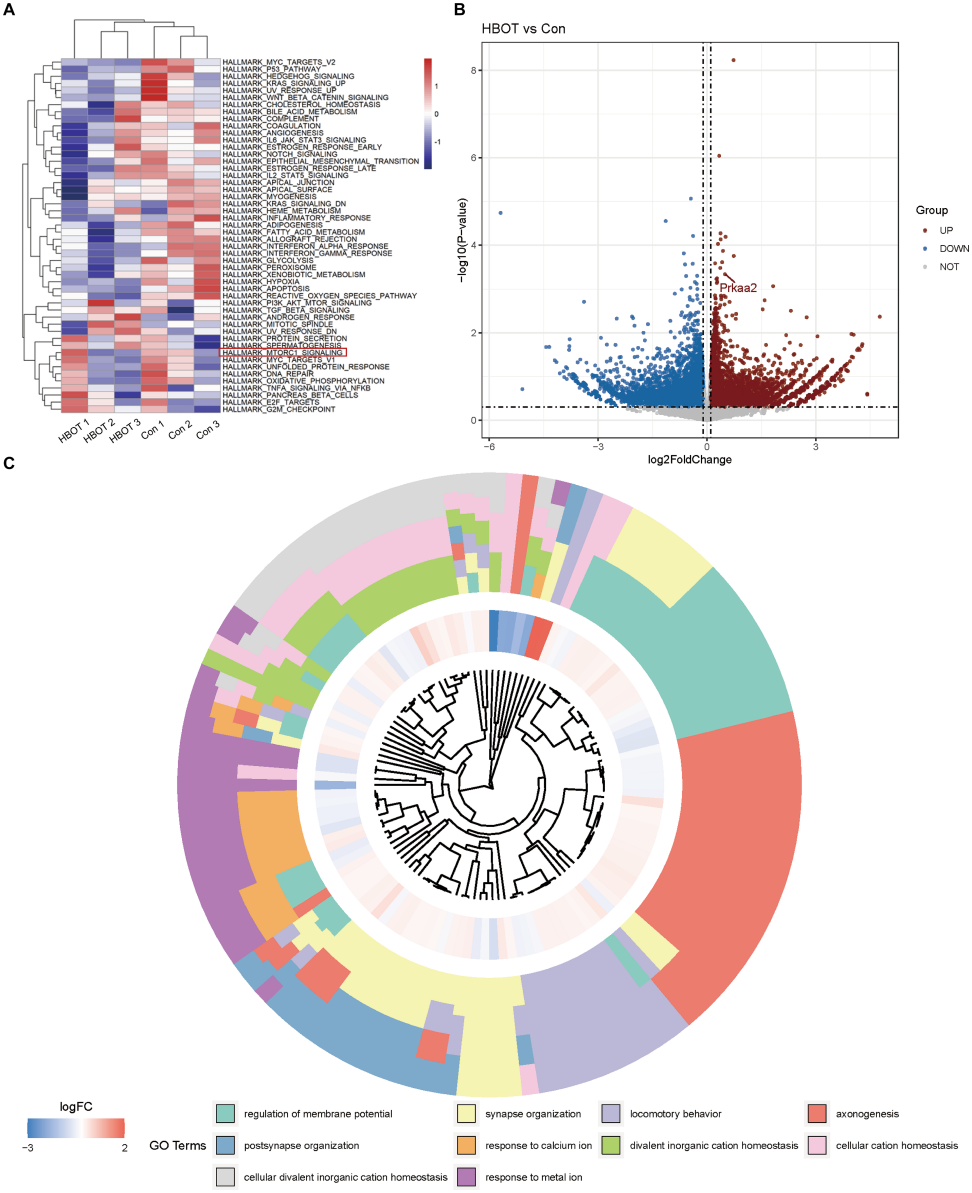
Bioinformatics analysis of eukaryotic referent transcriptome in aged C57BL/6 mice. **(A)** Gene Set Variation Analysis (GSVA) results of HBOT in aged C57BL/6 mice brain tissues. **(B)** Volcano plots showing the differentially expressed genes in HBOT vs. control. Prkaa2 is one of the upregulated genes (*p* < 0.05, log2FoldChange>0.1). **(C)** GO terms enrichment analyses of differently genes between in HBOT and control. The squares in the outer ring represent the GO terms. HBOT, hyperbaric oxygen therapy; Con, Control.

We presented the Gene Set Variation Analysis (GSVA) results of the aged C57BL/6 mouse brain tissues subjected to HBOT (Figure 3A). Our GSVA findings indicated that HBOT could downregulate several signaling pathways, including the MTORC1 pathway known for its inhibitory role in autophagy activation (Wan et al., 2018). Volcano plots demonstrating the DEGs when comparing the HBOT group to the control group were depicted in (Figure 3B). We observed an upregulation of the gene prkaa2, corresponding to the AMPKα2 protein. Finally, the Gene Ontology (GO) term enrichment analysis of differentially expressed genes between the HBO-treated and the control groups is shown in (Figure 3C). The squares in the outer ring represent the specific GO terms. This analysis showed that HBOT could notably upregulate several GO terms, including postsynapse organization, synapse organization, locomotory behavior, and axonogenesis. This further elucidates the molecular mechanisms through which HBO treatment exerts its effects.

### HBOT changed hippocampal microstructure in aged C57BL/6 mice

**Figure 4:**
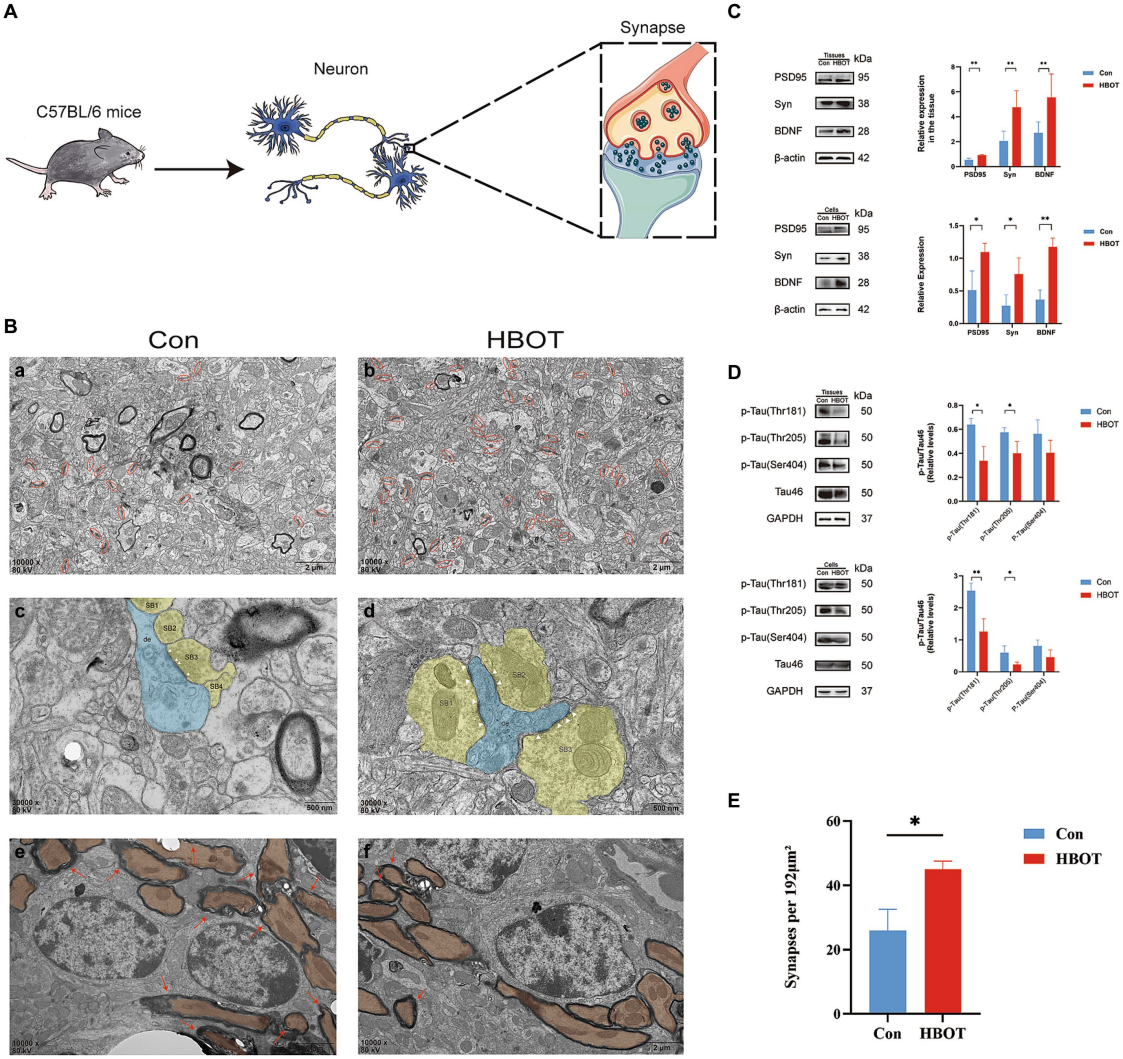
Effects of HBOT on neurocognitive measures in aged C57 BL/6 mice. **(A)** Diagram of neuron and synapse in aged C57BL/6 mice. **(B)** Synapse changes in hippocampus viewed with electron microscopy. **(A,B)** Synaptic boutons (yellow) on the dendrite (blue) with visible active zones (AZ, white arrowheads) in the HBOT group compared to control. **(C,D)** Fewer demyelinated lesions (red arrows) seen in the HBOT group. **(E,F)** Control group has more synaptic junctions (red circles) than HBOT group. Scale bars: **(a,b)** (500 nm); **(c–f)** (2 μm). **(C)** Increased levels of PSD95, BDNF and Syn proteins in aged mice and N2a cells post-HBOT (*n* = 3/group, *p < 0.05, **p < 0.01). Expression normalized to β-actin. **(D)** HBOT decreases p-Tau (Thr181, Thr205) levels but leaves Tau46 and p-Tau (Ser404) levels unchanged in aged mice and N2a cells (*n* = 3/group, *p < 0.05, **p < 0.01). Expression normalized to GAPDH. PSD, postsynaptic density; AZ, active zone; de, dendrite. **(E)** Statistical analysis of synaptic density. Compared to the Con group, the synaptic density (highlighted in red circles) in the HBOT group is significantly higher per 192 μm^2^ (*n* = 3/group, *p < 0.05).

Our findings demonstrate that HBOT significantly affects synaptic structures in the hippocampi of aged C57 BL/6 mice (Figure 4A). TEM was utilized to observe ultrastructural alterations in hippocampal synapses. Firstly, we noted a marked increase in synaptic number in the HBOT group compared to controls. This suggests that HBOT may exert a beneficial impact on synaptic plasticity, potentially ameliorating age-related synaptic loss (Figures 4B,D). Secondly, we observed changes in the active zone, an area of the presynaptic membrane where synaptic vesicles dock and fuse to release neurotransmitters (Jung, 2019). The active zone size in the HBOT group was notably larger than in the control group, indicating an enhancement of synaptic function due to the treatment. Finally, we identified a reduction in demyelinated lesions in the HBOT group compared to controls. These findings indicate that HBOT may facilitate remyelination or preserve myelin integrity in the aging brain (Figure 4B).

TEM analysis of hippocampal synapses further supported the positive effects of HBOT on synaptic integrity. Compared to the control group, the HBOT group exhibited improved synaptic morphology, including clear active zones and increased synaptic junctions. Additionally, the HBOT group showed fewer demyelinated lesions, indicating potential preservation of myelination in the hippocampus. These findings suggest that HBOT may positively impact synaptic integrity and myelination, which could contribute to improved neurocognitive function in aged mice.

Following 14 days of HBOT, we evaluated PSD95, BDNF, and Syn protein expression levels in aged C57BL/6 mice via Western blotting. We observed a marked increase in the expression of these proteins in the HBOT-treated group relative to the control group. This upregulation indicates an enhancement in synaptic plasticity and neurotrophic support, crucial factors in cognitive function and neural resilience. In a parallel experiment, we observed similar trends in N2a cells exposed to HBOT (Figure 4C). This consistency between *in vivo* and *in vitro* models further strengthens the evidence of HBOT’s potential impact on neuroplasticity and brain health.

Notably, the upregulation of these proteins may serve as a mechanism underlying the cognitive enhancement induced by HBOT. By facilitating synapse formation, stability, and transmission enhancement, HBOT promotes the maintenance and improvement of neural network function, potentially benefiting learning, memory, and cognitive processes.

### HBOT ameliorated tau hyperphosphorylation in aged C57BL/6 mice and N2a cells

We performed Western blot analysis to evaluate the phosphorylated tau (p-tau) and total tau protein levels in aged C57BL/6 mice following a 14-day HBOT regimen. The HBOT-treated group demonstrated a significant decrease in tau phosphorylation at Thr181 and Thr205 residues, while the total tau protein levels remained unaltered. This suggests that HBOT may selectively modulate tau phosphorylation without impacting the overall tau protein expression. Interestingly, the phosphorylation status of tau at the Ser404 residue did not show a significant reduction in the HBOT-treated group, indicating residue-specific effects of HBOT on tau phosphorylation. Similar patterns of tau phosphorylation were observed in N2a cells subjected to HBOT, corroborating the *in vivo* findings. This consistency across *in vivo* and *in vitro* models further strengthens the suggestion that HBOT may influence tau phosphorylation status, a critical factor implicated in neurodegenerative disorders. However, more extensive studies are required to elucidate further the exact mechanisms by which HBOT impacts tau biology and neurodegenerative processes (Figure 4D).

### HBOT induced an increase in autophagy

**Figure 5:**
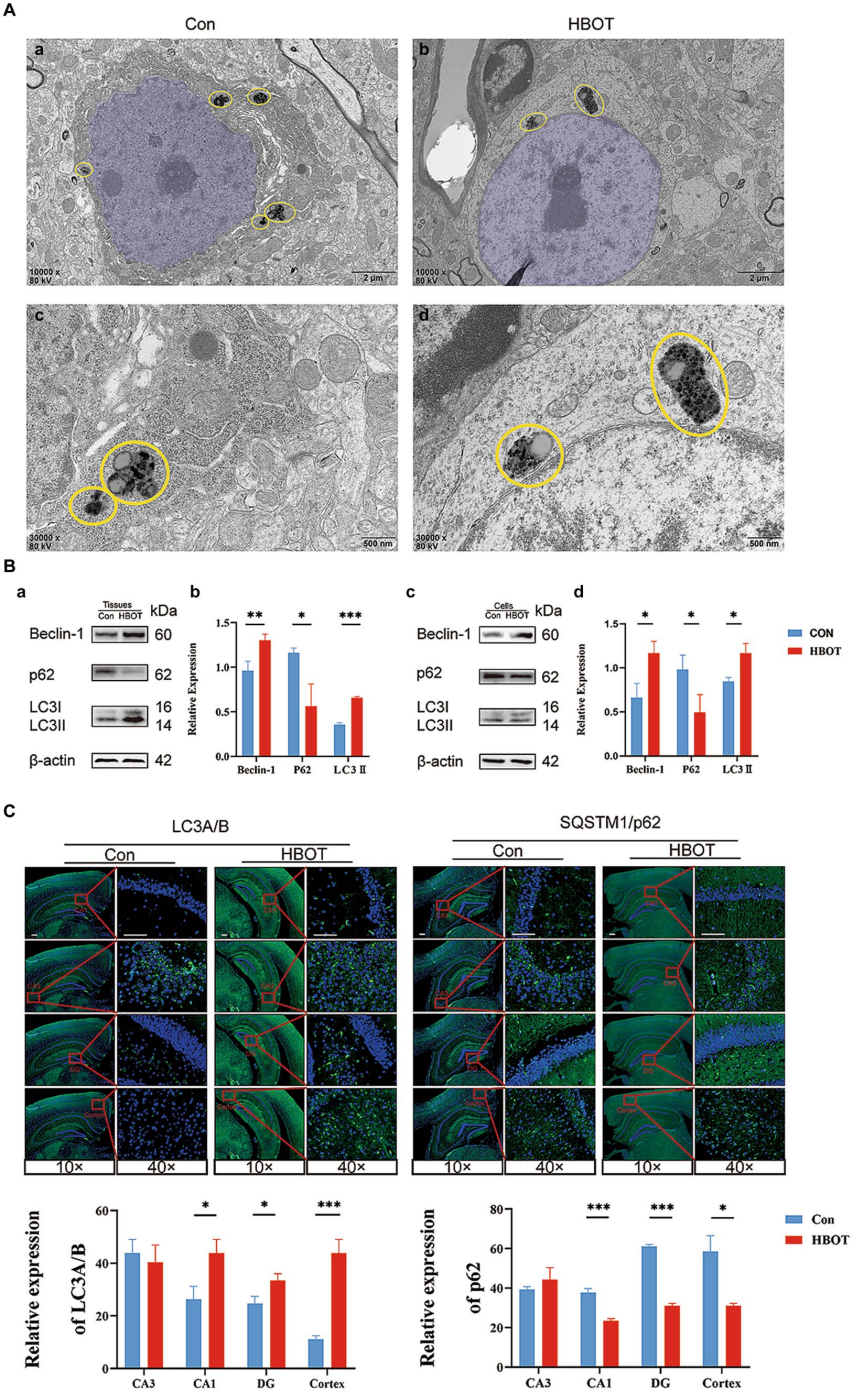
Impact of HBOT on autophagy function in aged C57BL/6 mice. **(A)** Comparison of neuronal structures in HBOT-treated and control groups. The neurons in the HBOT-treated group present a healthy structure, characterized by elliptically shaped nuclei (highlighted in light purple) with evenly distributed chromatin **(Aa,c)**. In contrast, neurons in the control group show irregular nuclear morphology and chromatin clustering **(Ab,d)**. Yellow circles highlight the “PANTHOS” structural configuration (Lee et al., 2022). The HBOT groups exhibited a lower incidence of PANTHOS compared to the Control groups. **(B)** Impact of HBOT on autophagy-related proteins. HBOT increases Beclin-1 and LC3 expression and decreases p62 expression in aged mice and N2a cells (*n* = 3/group, *p < 0.05, **p < 0.01, ***p < 0.001). Expression normalized to β-actin. **(C)** Visualization of LC3A/B and p62 expression in the hippocampus and cortex via immunofluorescence. Scale bars = 100 μm (*n* = 3/group, *p < 0.05, **p < 0.01, ***p < 0.001).

Our results demonstrated the impact of HBOT on neuronal integrity and the expression of autophagy-related proteins in aged C57BL/6 mice. Observations from TEM revealed neurons in the HBOT-treated group maintained an intact structure characterized by an elliptical nucleus, evenly distributed chromatin, and an intact nuclear membrane. On the other hand, neurons from the control group exhibited a slight shrinkage in the cell body, chromatin aggregation in the nucleus, and an intact nuclear membrane (as shown in Figures 5A,B). We identified a distinctive structural arrangement named “PANTHOS” in the electron microscopy images. The PANTHOS configuration can be considered a unique manifestation of autophagy dysfunction in Alzheimer’s disease (Lee et al., 2022). Notably, the instances of PANTHOS were significantly reduced in the HBOT group compared to the control group (Figure 5A).

Western blot analyses were also conducted in our study. We found that HBOT elevated the levels of Beclin-1 and LC3 proteins and reduced the level of p62 protein in the hippocampus region of the aged mice (Con, *n* = 3; HBOT, *n* = 3). Similar trends were observed when N2a cells were exposed to HBOT, corroborating our findings. The relative protein expression was normalized to β-actin, underscoring the significant differences (Figure 5B) (*p < 0.05, **p < 0.01, ***p < 0.001).

In addition, Immunofluorescence staining revealed alterations in the expression of autophagy-associated proteins LC3A/B and p62 within the hippocampus and cortex, with an elevated level of LC3 expression and a decreased level of p62 observed following HBOT. The observable changes, visualized with scale bars equal to 100 μm, reaffirm the potential of HBOT in modulating autophagy-related processes (Figure 5C).

### HBOT reduced the expression of brain senescence markers

**Figure 6:**
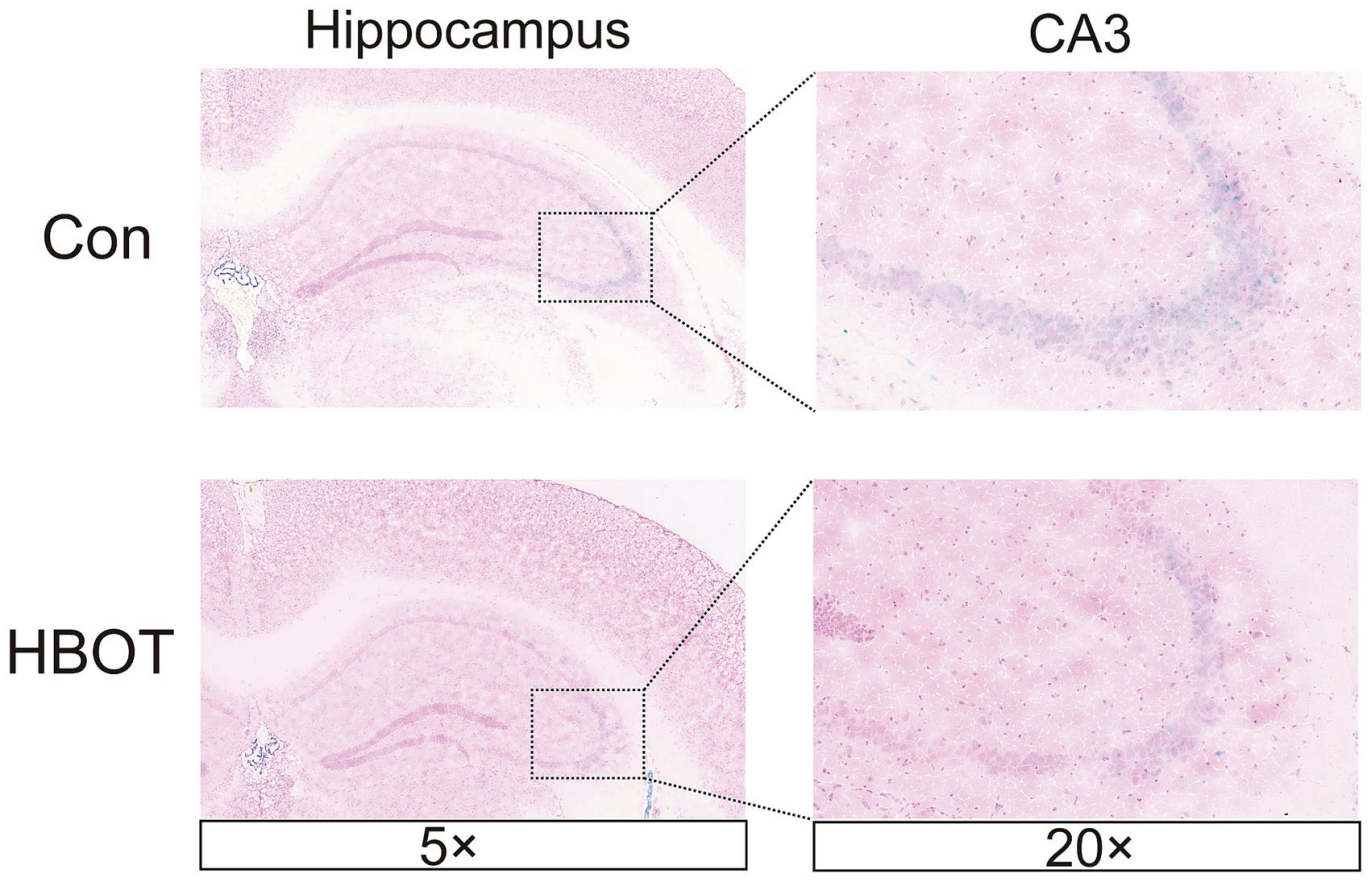
HBOT alleviates hippocampal cell senescence. Representative images of SA-β-Gal+ in HBOT group mice and Control group mice in the hippocampus. The enlarged boxed region shows the region CA3-positive cells. Scale bars = 50 μm.

Senescence-associated β-galactosidase (SA-β-Gal) is a widely used indicator of cellular senescence (Chen et al., 2009). This study employed SA-β-Gal staining to assess brain senescence by examining tissue samples. Our findings reveal a notable reduction in the quantity of SA-β-Gal-positive cells within the mouse hippocampus following HBOT treatment, particularly in the CA3 region, compared to the control group (Figure 6).

### HBOT activated the AMPK-mTOR signaling pathway, a critical regulator of autophagy

**Figure 7:**
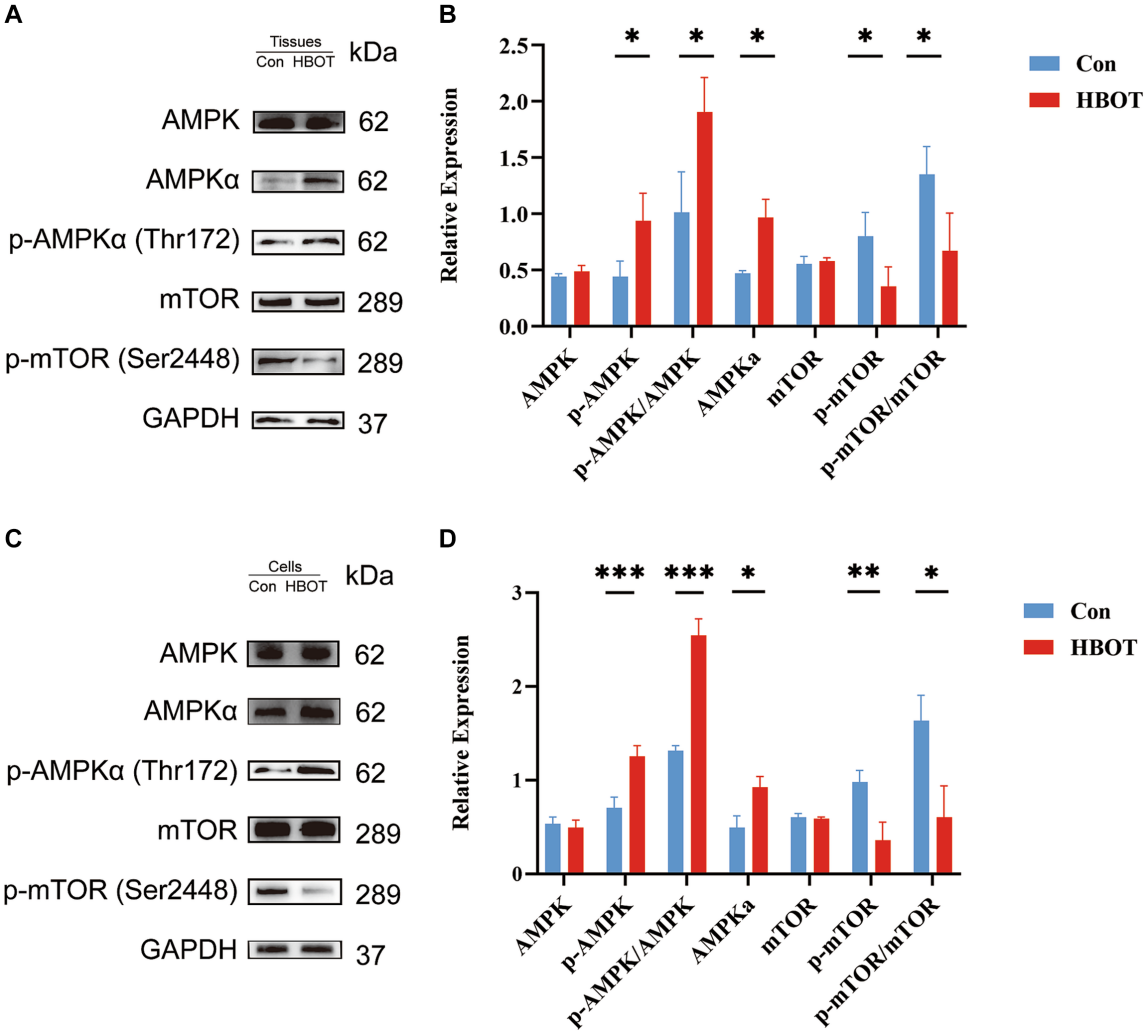
Impact of HBOT on the protein levels of the AMPK/mTOR pathway. Representative immunoblot bands for m-TOR, p-m-TOR, AMPKα, p-AMPKα in the hippocampal region of the control group and the HBOT group mice are presented (as shown in **A**). Statistical analyses demonstrated that HBOT increased the level of p-AMPKα and the ratio of p-AMPKα/AMPKα, and decreased the level of p-mTOR and the ratio of p-mTOR/mTOR in the hippocampal region (as shown in **B**). Similar results were observed when N2a cells were treated with HBOT **(C,D)**. (*n* = 3/group, *p < 0.05, **p < 0.01, ***p < 0.001).

We performed a Western blot analysis to verify whether the augmentation of autophagy induced by HBOT depends on regulating the AMPK-mTOR signaling pathway. HBOT augmented the level of p-AMPKα and the ratio of p-AMPKα/AMPKα and decreased the level of p-mTOR as well as the ratio of p-mTOR/mTOR within the hippocampal region (Con, *n* = 3; HBOT, *n* = 3) (Figures 7A,B). These observations were echoed when N2a cells were subjected to HBOT, exhibiting similar trends (Figures 7C,D). As such, this revealed an elevation in the level of phosphorylated AMPK (Thr172) and a reduction in the level of phosphorylated mTOR (Ser2448), indicating that HBOT activates the AMPK-mTOR signaling pathway, a key regulator of autophagy.

## Discussion

In this study, we investigated the effects of HBOT on cognitive function, tau phosphorylation, synaptic function, and autophagy in aged C57BL/6 mice. We have also made intriguing observations regarding the influence of HBOT on cellular aging processes, including the downregulation of critical markers associated with senescence, such as SA-β-gal. The results provide valuable insights into the potential mechanisms underlying the cognitive-enhancing effects of HBOT and its impact on molecular pathways related to aging and age-related diseases.

Our research revealed that HBOT markedly improves spatial memory and learning capabilities in elderly mice. In a randomized controlled trial involving 63 elderly individuals, researchers observed that a daily 60-min HBOT regimen led to cognitive enhancements in attention, information processing speed, and executive functions. This improvement is possibly attributed to an upsurge in regional cerebral blood flow (Hadanny et al., 2020). Some studies also show that HBOT can improve the cognitive and behavioral function of children with persistent post-concussion syndrome (PPCS) in the chronic phase (Hadanny et al., 2022).

So, how does HBOT enhance cognitive function? Hadanny et al. proposed intermittent exposure to high oxygen levels can induce many cellular and molecular mechanisms typically triggered by hypoxia. This phenomenon is referred to as the Hyperoxic-Hypoxic Paradox (HHP). In clinical practice, HBOT can generate intermittent high levels of oxygen. HBOT can induce cellular cascades through hypoxia-related factors such as hypoxia-inducible factors (HIF), vascular endothelial growth factor (VEGF), and Sirtuin. Additionally, it can increase the mobilization of circulating stem cells and promote mitochondrial biogenesis (Hadanny and Efrati, 2020). Although a precise dose–response curve has not been established in clinical practice, specific HBOT protocols have demonstrated the ability to induce tissue regeneration in damaged tissues (Hadanny and Efrati, 2020). However, a crucial observation in our study is the intriguing association between HBOT and autophagy, an essential cellular process tasked with clearing damaged proteins and organelles. Disabled autophagy is a significant hallmark of aging. “PANTHOS” refers to a unique pathological pattern of autophagy dysfunction observed in the brains of Alzheimer’s disease patients, characterized by an abundant accumulation of Aβ-positive autophagic vesicles that form large membrane-bound vesicles, creating a flower-shaped perinuclear ring (Lee et al., 2022). Our research findings suggested that HBOT can significantly decrease the numbers of PANTHOS in the cytoplasm. This may indicate that HBOT can potentially improve or rectify the impairment of autophagy function. Stimulation of autophagic flux has been broadly evidenced to enhance health span and lifespan in model organisms (López-Otín et al., 2023). Our study revealed that HBOT might modulate degradation and recycling mechanisms. Thus, it is plausible to conclude that HBOT could activate autophagy based on the observed changes in autophagy-related proteins. Beclin-1 is a crucial player in the initial stages of autophagy, and LC3 is an essential component of autophagosome formation and maturation. An increase in Beclin-1 and LC3 expression suggests the enhanced initiation and progression of autophagy. Meanwhile, p62 is a protein that binds to LC3 and guides cargo (damaged or unnecessary cellular components) to the autophagosome for degradation. As p62 is also degraded during autophagy, a decrease in p62 levels typically signifies active or increased autophagic flux (Hirano et al., 2016; Chen et al., 2019). Our study showed that HBOT increases Beclin-1 and LC3 expression and decreases p62 expression, which suggests that HBOT induces or enhances the autophagic process. This induction of autophagy could potentially contribute to improved cognitive function in aged mice by removing accumulated, damaged cellular components, reducing oxidative stress, and maintaining cellular homeostasis. It may even play a role in decelerating aging.

Consistent with our research, another study also demonstrated that HBOT treatment increases the expression of autophagy-related markers, such as LC3-II and Beclin-1, which confirmed that HBOT enhanced autophagy activity (Yan et al., 2011). Besides, HBOT has been shown to enhance autophagy in the brain, which may contribute to the clearance of toxic protein aggregates and damaged cellular components (Fang et al., 2019). The induction of autophagy by HBOT may protect neuronal cells from oxidative stress, inflammation, and other pathological processes, thereby preserving cognitive function (Lin et al., 2012; Stetler et al., 2014). In addition to its effects on the brain, HBOT has also been reported to modulate autophagy in other tissues. Furthermore, an added study also demonstrated that HBOT promotes autophagy in tissue repair and wound-healing cells, such as fibroblasts and endothelial cells (Yang et al., 2017). This autophagy induction may aid in removing cellular debris, enhance cell survival, and facilitate tissue regeneration processes. Therefore, these findings suggest that HBOT can activate autophagy in different cell types and tissues.

One potential mechanism by which HBOT activates autophagy is through signaling pathway modulation. One study showed that HBOT can effectively relieve neuropathic pain by activating autophagic flow and inhibiting the mTOR pathway (Liu et al., 2017). It has also been reported that HBOT can activate the AMPK pathway, a key regulator of autophagy. Activation of AMPK inhibits the mTOR pathway, which is a negative regulator of autophagy, thus promoting autophagic activity (Alers et al., 2012; Russell et al., 2014; Menzies et al., 2017). In our current study, we observed that HBOT increased the level of p-AMPK and decreased the level of p-mTOR in the hippocampal region, which implies that the activation of the AMPK/mTOR-mediated autophagy pathway may be involved in the cognitive improvement observed in our study. However, more studies are required to confirm this hypothesis and further elucidate the specific mechanistic pathways involved.

Our study also presented an in-depth investigation of the specific influences of HBOT on brain synapses in aging mice. Transcriptomic analyses revealed significant alterations in gene expression profiles, particularly in modulating synaptic function and plasticity, following HBOT. Moreover, we found HBOT to enhance synaptic structures in the hippocampi of aged mice by transmission electron microscopy, potentially counteracting age-related synaptic loss. A recent study also showed that HBOT contributes to the suppression of dendritic and synaptic degeneration and apoptosis, which indicates that the beneficial effects of HBOT may be associated with its ability to maintain or improve synaptic integrity (Ying et al., 2019). Another study indicated that HBOT can enhance neural plasticity responses by promoting axonal growth and synaptic remodeling, thus restoring motor function in rats with traumatic brain injury (Brkic et al., 2012). Furthermore, our data indicated increased PSD95, BDNF, and Syn protein expression and reduced tau protein phosphorylation following HBOT, suggesting a potential impact on neurodegenerative diseases. Recent research indicated that HBOT may reduce tau protein phosphorylation by decreasing hypoxic conditions and neuroinflammation (Shapira et al., 2018), consistent with our findings.

A critical avenue for future research involves exploring the enduring impacts of HBOT on cognitive performance, initially in various animal models and ultimately in human participants (Lin et al., 2012). Although our study offers encouraging outcomes on the cognitive enhancement capabilities of HBOT in elderly mice, it is crucial to corroborate these results in additional animal models, such as rats or non-human primates. This step is vital to reinforce the therapeutic applicability of HBOT for human use. Additionally, conducting clinical trials to evaluate the efficacy and safety of HBOT in improving cognitive function in elderly individuals or those with age-related neurodegenerative diseases is warranted. Moreover, exploring the optimal treatment duration, frequency, and pressure levels of HBOT is crucial. Our study employed a 14-day HBOT protocol, but it is critical to determine whether shorter or longer treatment durations or different treatment regimens can yield even more pronounced cognitive improvements. Furthermore, investigating the potential synergistic effects of combining HBOT with other therapeutic interventions, such as pharmacological agents or cognitive training, may enhance the therapeutic outcomes and provide a comprehensive approach to combat cognitive impairment. Thus, future studies should aim to elucidate the underlying molecular mechanisms by which HBOT exerts cognitive-enhancing effects. Investigating the specific signaling pathways, gene expression profiles, and epigenetic modifications involved in HBOT-1-induced cognitive improvements can provide valuable insights into the intricate molecular networks underlying cognitive function and age-related neurodegeneration. Therefore, understanding the interactions between HBOT, the AMPK-mTOR pathway, autophagy, and other cellular processes involved in neuronal homeostasis and plasticity is instrumental in developing targeted therapeutic interventions for cognitive dysfunction.

## Conclusion

**Figure 8:**
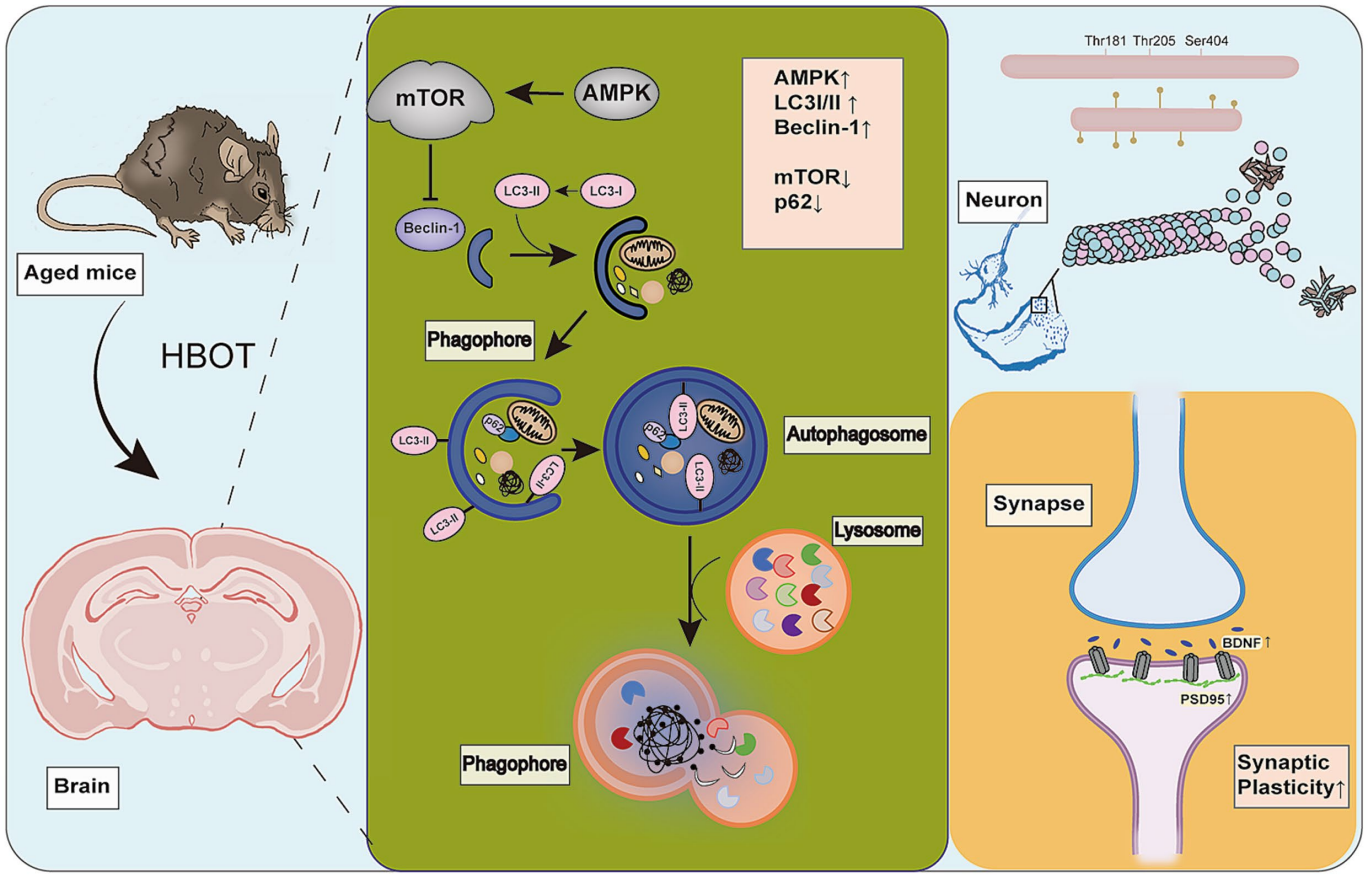
HBOT Mechanism. HBOT increases cellular autophagy, leading to upregulation of AMPK, beclin-1, and LC3I/II proteins. It also downregulates mTOR and p62 proteins. HBOT inhibits phosphorylation of tau protein (Thr181, Thr205) and improves synaptic plasticity by increasing levels of Syn, BDNF, and PSD95 proteins.

In summary, we employed a comprehensive experimental design to find that HBOT potentially improves the spatial memory and learning capabilities, synaptic integrity, and tau phosphorylation in aged mice, potentially by activating autophagy via the AMPK-mTOR pathway, thereby enhancing their cognitive functions (Figure 8). These results highlight the importance of synaptic plasticity, tau pathology, and cellular clearance mechanisms in cognitive function. This also suggests that HBOT may be a promising therapeutic strategy for aging-related cognitive decline and neurodegenerative disorders. To some extent, our research fills the knowledge gap regarding the impact of HBOT on the aging brain. However, further studies are necessary to elucidate how HBOT impacts the aging brain at the molecular and cellular levels and determine these findings’ applicability in a broader population.

## Data availability statement

The data presented in the study are deposited in the NCBI repository, Accession to cite for these SRA data: PRJNA1071211. The direct link is: URL: https://www.ncbi.nlm.nih.gov/sra/PRJNA1071211.

## Ethics statement

The animal study was approved by the Research Ethics Committee of Chongqing General Hospital. The study was conducted in accordance with the local legislation and institutional requirements.

## Author contributions

SW: Conceptualization, Data curation, Formal analysis, Investigation, Methodology, Project administration, Software, Writing – original draft, Writing – review & editing. BC: Writing – review & editing, Data curation, Investigation, Methodology. MY: Data curation, Writing – review & editing, Formal analysis, Software, Supervision. SL: Data curation, Formal analysis, Investigation, Methodology, Project administration, Writing – original draft. HF: Data curation, Formal analysis, Writing – review & editing. XY: Data curation, Writing – review & editing. QZ: Data curation, Writing – review & editing. YP: Data curation, Writing – review & editing. ZC: Conceptualization, Funding acquisition, Resources, Supervision, Validation, Visualization, Writing – review & editing.

## Footnotes

^1^ https://www.bioinformatics.babraham.ac.uk/projects/fastqc/

^2^ www.bioconductor.org

^3^ http://www.genome.jp/kegg/
